# A Polygenic Risk Score Derived From Episodic Memory Weighted Genetic Variants Is Associated With Cognitive Decline in Preclinical Alzheimer’s Disease

**DOI:** 10.3389/fnagi.2018.00423

**Published:** 2018-12-19

**Authors:** Tenielle Porter, Samantha C. Burnham, Greg Savage, Yen Ying Lim, Paul Maruff, Lidija Milicic, Madeline Peretti, David Ames, Colin L. Masters, Ralph N. Martins, Stephanie Rainey-Smith, Christopher C. Rowe, Olivier Salvado, Kevin Taddei, David Groth, Giuseppe Verdile, Victor L. Villemagne, Simon M. Laws

**Affiliations:** ^1^Collaborative Genomics Group, Centre of Excellence for Alzheimer’s Disease Research and Care, School of Medical and Health Sciences, Edith Cowan University, Joondalup, WA, Australia; ^2^Cooperative Research Centre (CRC) for Mental Health, Carlton, VIC, Australia; ^3^CSIRO Health and Biosecurity, Parkville, VIC, Australia; ^4^Centre of Excellence for Alzheimer’s Disease Research and Care, School of Medical and Health Sciences, Edith Cowan University, Joondalup, WA, Australia; ^5^ARC Centre of Excellence in Cognition and its Disorders, Department of Psychology, Macquarie University, North Ryde, NSW, Australia; ^6^The Florey Institute of Neuroscience and Mental Health, The University of Melbourne, Parkville, VIC, Australia; ^7^CogState Ltd., Melbourne, VIC, Australia; ^8^Academic Unit for Psychiatry of Old Age, St. Vincent’s Health, The University of Melbourne, Kew, VIC, Australia; ^9^National Ageing Research Institute, Parkville, VIC, Australia; ^10^Department of Nuclear Medicine and Centre for PET, Austin Health, Heidelberg, VIC, Australia; ^11^School of Pharmacy and Biomedical Sciences, Faculty of Health Sciences, Curtin Health Innovation Research Institute, Curtin University, Bentley, WA, Australia

**Keywords:** polygenic risk score, Alzheimer’s disease, Aβ-amyloid, cognitive decline, episodic memory

## Abstract

Studies of Alzheimer’s disease risk-weighted polygenic risk scores (PRSs) for cognitive performance have reported inconsistent associations. This inconsistency is particularly evident when PRSs are assessed independent of *APOE* genotype. As such, the development and assessment of phenotype-specific weightings to derive PRSs for cognitive decline in preclinical AD is warranted. To this end a episodic memory-weighted PRS (*em*PRS) was derived and assessed against decline in cognitive performance in 226 healthy cognitively normal older adults with high brain Aβ-amyloid burden participants from the Australian Imaging, Biomarkers and Lifestyle (AIBL) study. The effect size for decline in a verbal episodic memory was determined individually for 27 genetic variants in a reference sample (*n* = 151). These were then summed to generate a *em*PRS either including *APOE* (*em*PRS^$\bar{c}$*APOE*^) or excluding *APOE* (*em*PRS^$\bar{s}$*APOE*^). Resultant *em*PRS were then evaluated, in a test sample (*n* = 75), against decline in global cognition, verbal episodic memory and a pre-Alzheimer’s cognitive composite (AIBL-PACC) over 7.5 years. The mean (SD) age of the 226 participants was 72.2 (6.6) years and 116 (51.3%) were female. Reference and test samples did not differ significantly demographically. Whilst no association of *em*PRSs were observed with baseline cognition, the *em*PRS^$\bar{c}$*APOE*^ was associated with longitudinal global cognition (-0.237, *P* = 0.0002), verbal episodic memory (-0.259, *P* = 0.00003) and the AIBL-PACC (-0.381, *P* = 0.02). The *em*PRS^$\bar{s}$*APOE*^ was also associated with global cognition (-0.169, *P* = 0.021) and verbal episodic memory (-0.208, *P* = 0.004). Stratification by *APOE* ε4 revealed that the association between the *em*PRS and verbal episodic memory was limited to carriage of no ε4 or one ε4 allele. This was also observed for global cognition. The *em*PRS and rates of decline in AIBL-PACC were associated in those carrying one ε4 allele. Overall, the described novel *em*PRS has utility for the prediction of decline in cognition in preclinical AD. This study provides evidence to support the further use and evaluation of phenotype weightings in PRS development.

## Introduction

An improved understanding of the extended preclinical phase of Alzheimer’s disease (AD), has seen an increased focus on early disease intervention (Sperling et al., 2014). As a result, importance has been placed on investigating potential factors that could underpin the significant variability in cognitive decline between individuals in this early stage of the disease. Accumulation of Aβ-amyloid (Aβ) occurs up to 20 years prior to symptom onset (Villemagne et al., 2013). In addition, abnormally high neocortical Aβ in cognitively normal (CN) older adults is associated with an increased risk for cognitive decline and development of AD (Villemagne et al., 2011). Despite this, levels of Aβ alone do not track well with progressive cognitive decline and there is strong convergent evidence that variable rates of decline in the preclinical stages of AD may be influenced by genetic factors (Lim et al., 2015a,b; Porter et al., 2018a,c). Identification of genetic factors that contribute to accelerated rates of cognitive decline in at risk individuals will be of significant importance, through an increased understanding of potential mechanisms of preclinical decline and the identification of individuals most suitable for intervention trials.

One method frequently investigated for use as a predictor of cognitive performance and decline employs polygenic risk scores (PRSs). These are typically focused on AD risk associated genes identified through genome wide association studies (GWAS). Once identified, these genetic variants are weighted by their respective effect sizes and summed. The resulting scores have then been used for the analysis of associations with clinical and pathological variables including: measures of clinical classification (Biffi et al., 2010; Rodriguez-Rodriguez et al., 2013; Chauhan et al., 2015; Escott-Price et al., 2015, 2017; Sleegers et al., 2015; Chouraki et al., 2016; Mormino et al., 2016; Desikan et al., 2017; Lacour et al., 2017), disease progression, and fluid (Sabuncu et al., 2012; Martiskainen et al., 2015; Sleegers et al., 2015; Louwersheimer et al., 2016) and imaging (Biffi et al., 2010; Sabuncu et al., 2012; Chauhan et al., 2015; Habes et al., 2016; Harrison et al., 2016; Lupton et al., 2016; Mormino et al., 2016; Foley et al., 2017) biomarkers. However, inconsistent findings have been reported when investigating associations of PRSs with cognition. Almost equally, studies have observed significant associations with cognitive performance (Sabuncu et al., 2012; Carrasquillo et al., 2015; Andrews et al., 2016; Louwersheimer et al., 2016; Marden et al., 2016; Mormino et al., 2016) or an absence of association (Gui et al., 2014; Harrison et al., 2016; Bressler et al., 2017; Darst et al., 2017).

We have previously reported that while an AD risk weighted PRS was associated with cognitive decline (Porter et al., 2018b), the association was only observed in carriers of the apolipoprotein E (*APOE*) ε4 allele. This is consistent with other studies which have reported no association with cognition when *APOE* was removed from the calculation of a PRS (Carrasquillo et al., 2015; Andrews et al., 2016). In addition to being the strongest known genetic risk factor for the development of AD, the ε4 allele of *APOE* has previously been associated with cross-sectional and longitudinal cognitive performance (Lim et al., 2015b). Of particular interest, carriage of *APOE* ε4 in CN older adults at risk for AD (determined by Aβ brain imaging) is associated with accelerated decline in multiple cognitive domains (Lim et al., 2015b).

In addition to *APOE*, a number of genetic variants with no or limited association with AD risk have been independently associated with cognitive performance in diseased, at risk and healthy populations. The genes containing these variants have roles in promotion of neuronal survival (*BDNF*; Brain Derived Neurotropic Factor; (Lim et al., 2015a), synaptic plasticity (*KIBRA*; Kidney and Brain expressed protein; (Tracy et al., 2016; Porter et al., 2018a), regulation of dopamine availability (*COMT*; Catechol-*O*-methyltranferase; Sheldrick et al., 2008), longevity (*KL*; Klotho; Arking et al., 2002), inflammation (*CSMD1*; CUB and Sushi Multiple Domain 1; Kraus et al., 2006) and amyloid precursor protein (APP) processing (*SPON1*; Spondin 1; Ho and Sudhof, 2004). In addition to the independent association of these genes with cognitive performance, we have also recently reported on the utility of combining these cognition-associated genetic variants for assessing longitudinal cognition (Porter et al., 2018c). As identified above there is also a significant body of literature combining GWAS derived AD risk associated genetic variants, typically within a core set of 21 genes, into PRSs that have previously been associated with the clinical classification of AD and disease phenotypes, albeit with inconsistency of association with cognition. However, there are few studies that have investigated the combination of AD risk and cognition associated variants. This is likely due the possible dilution of the effects of the cognition associated genetic variants when their weak AD risk weightings are applied (Andrews et al., 2016).

Reasons for the inconsistency of studies investigating PRSs may be twofold. First, cognition has significant inter-individual variability, particularly in the elderly, which works to increase the difficulty of predicting rates of cognitive decline. Second, late disease stage methods of weighting, such as AD risk, may not be suitable for predicting cognitive decline at early preclinical stages. As such, a cognitive phenotype, such as verbal episodic memory, may be more appropriate as it is typically observed to precede decline in executive function by 4–8 years and between 7 and 10 years before other domains (Elias et al., 2000; Grober et al., 2008; Derby et al., 2013). The hypothesis of this study was therefore: through combining individually weighted AD risk and cognitive decline associated genetic variants, by endophenotype effect-sizes, a PRS can be derived with utility for prediction of preclinical rates of cognitive decline. Further, by focusing on CN older adults with high levels of neocortical Aβ, population heterogeneity, and so cognitive performance variability, is reduced. To test this hypothesis, the study undertook a targeted approach to the development and assessment of utility of a novel episodic memory-weighted PRS (*em*PRS), by weighting each genetic variant by its effect on decline in verbal episodic memory, in CN older adults with high neocortical Aβ burden.

## Materials and Methods

### Study Participants

Data is reported on 232 CN older adults enrolled in the Australian Imaging Biomarkers and Lifestyle (AIBL) Study of Ageing. The AIBL Study is a prospective longitudinal study of aging, the study design, enrolment process, neuropsychological assessments, and diagnostic criteria have been previously described (Ellis et al., 2009). Briefly, a participant was classified by a clinical review panel (Ellis et al., 2009), blinded to Aβ-amyloid status, as CN if they did not meet the clinical criteria for diagnosis of mild cognitive impairment (MCI) (Winblad et al., 2004) or dementia (McKhann et al., 1984). Ethics approval was granted for the study by each member institution, including Austin Health, Edith Cowan University, Hollywood Private Hospital, and St Vincent’s Health. All participants provided informed written consent.

### Cognitive Measures

The AIBL neuropsychological test battery consists of the Mini-Mental State Examination (MMSE), Clock Drawing Test, California Verbal Learning Test-Second edition (CVLT-II), Logical Memory I and II (LMI; LMII; Story A only), D-KEFS verbal fluency, a 30-item version of the Boston Naming Test (BNT), Wechsler Test of Adult Reading (WTAR), Digit Span and Digit Symbol-Coding subtests of the Wechsler Adult Intelligence Scale-Third edition (WAIS-III), the Stroop task (Victoria version), and the Rey Complex Figure Test (RCFT) (Ellis et al., 2009). Test results were used in combination to calculate cognitive composite scores, as previously described (Donohue et al., 2014; Burnham et al., 2015, 2016). Specifically, in this study these composite scores included a measure of global cognition (CDR sum of boxes (CDR_SB_), MMSE, LMII, CVLT-II recognition false positives (CVLT-II_FP_) and Clock) and verbal episodic memory (CDR_SB_, LMII, CVLT-II_FP_) (Burnham et al., 2015), in addition to a composite of tests shown to be sensitive to decline in preclinical AD [AIBL-pre-Alzheimer’s cognitive composite (PACC); CVLT-II_LDFR_, LMII, MMSE, WAIS- III_DS-C_] (Donohue et al., 2014; Burnham et al., 2016). The calculation of the aforementioned composites involved corrections for age, sex, years of education, premorbid IQ [WTAR-estimated WAIS-III Full Scale Intelligence Quotient (FSIQ)] and depressive symptoms [Geriatric Depression Scale (GDS)] (Donohue et al., 2014). The AIBL-PACC did not include an age correction, however, this was included as a covariate in subsequent analyses. 7.5 years of cognitive assessment data was utilized with collections occurring at 0, 18, 36, 54, 72, and 90 months.

### Amyloid Imaging

All participants were imaged for neocortical Aβ by positron emission tomography (PET) using one of the following radiolabelled tracers; ^11^C-Pittsburgh Compound B (PiB), ^18^F-florbetapir or ^18^F-flutemetamol, as previously described (Rowe et al., 2010; Vandenberghe et al., 2010; Clark et al., 2011). CapAIBL^®^, a web-based, freely available software, was used to generate PET standardized uptake value (SUV) ratios (SUVR) for all tracers without the requirement for magnetic resonance imaging (Bourgeat et al., 2015). Target-region to reference-region SUVRs were calculated by the summation and normalization of SUVs to brain regions specific to each tracer [PiB (cerebellar cortex), florbetapir (whole cerebellum), flutemetamol (pons)]. All participants included in this study were classified as having a high Aβ (Aβ*^high^*) burden at any time point, as determined by tracer specific thresholds of ≥1.4, ≥1.05 and ≥0.55 for PiB, florbetapir and flutemetamol respectively.

### SNP Selection and Genotyping

A thorough literature review was conducted in PubMed and 27 single nucleotide polymorphisms (SNPs) were selected based on *a priori* evidence of associations with either AD risk or cognitive performance (or both; Table 1). Of these, 21 variants had previously been associated with the clinical classification of AD and disease phenotypes. The remaining six variants had previously been associated with cross-sectional and longitudinal cognitive phenotypes in cognitively normal and/or demented individuals. QIAamp DNA Blood Maxi Kits (Qiagen, Hilden, Germany) were used for the extraction of DNA from 5 mL of whole blood. TaqMan^®^ assays with the TaqMan^®^ GTXpress^TM^ Master Mix (Life Technologies) were used to genotype *APOE* (rs7412, assay ID: C____904973_10; rs429358, assay ID: C___3084793_20; Life Technologies, Carlsbad, CA, United States) on a QuantStudio 12K Flex^TM^ Real-Time-PCR system (Applied Biosystems, Foster City, CA, United States). QIAamp and TaqMan^®^ kits detailed above were used following manufacturer’s instructions. Genotype information for the additional SNPs included in the PRS were extracted from a genome-wide SNP array conducted on the Illumina OmniExpressHumanExome+ BeadChip with subsequent imputation using impute2 ver2.3, with the 1000 genome reference panel (2015 release). Complete SNP information was available for the 232 individuals included in the study. However, 6 samples were excluded from further analysis due to homozygosity of *KL*-VS variant, which has been reported to confer phenotypic risk not in a gene dosage dependent fashion (Arking et al., 2003). Analysis of all SNPs was performed using the dominant model of minor allele (Table 1).

**Table 1:** Single nucleotide polymorphisms (SNP) information.

Gene	SNP	Chromosome	Position	Minor allele	Risk genotype	AIBL Effect Size *d* (95% CI)
*APOE*	rs7412 rs429358	19	44908822 44908684	ε4	ε4+	0.472 (0.14 – 0.80)
*CR1*	rs3818361	1	207611623	A	G/G	0.109 (-0.24 – 0.46)
*BIN1*	rs744373	2	127137039	G	A/A	0.003 (-0.32 – 0.33)
*INPP5D*	rs35349669	2	233159830	T	T+	0.153 (-0.20 – 0.50)
*KIBRA*	rs17070145	5	168418786	C	C/C	0.097 (-0.22 – 0.42)
*MEF2C*	rs190982	5	88927603	G	A/A	0.278 (-0.07 – 0.62)
*HLA cluster*	rs9271192	6	32610753	C	C+	0.256 (-0.07 – 0.58)
*CD2AP*	rs9349407	6	47485642	C	C+	0.114 (-0.21 – 0.43)
*NME8*	rs2718058	7	37801932	G	G+	0.041 (-0.28 – 0.36)
*ZCWPW1*	rs1476679	7	100406823	C	T/T	0.149 (-0.17 – 0.47)
*EPHA1*	rs11767557	7	143412046	C	T/T	0.061 (-0.27 – 0.39)
*CSMD1*	rs2740931	8	4022021	A	A/A	0.201 (-0.16 – 0.56)
*CLU*	rs11136000	8	27607002	T	T+	0.078 (-0.25 – 0.40)
*PTK2B*	rs28834970	8	27337604	C	C+	0.030 (-0.30 – 0.36)
*SPON1*	rs11023139	11	14202800	A	A+	0.045 (-0.44 – 0.53)
*BDNF*	rs6265	11	27658369	Met	Met+	0.143 (-0.20 – 0.49)
*CELF1*	rs10838725	11	47536319	C	C+	0.103 (-0.22 – 0.42)
*MS4A6A*	rs610932	11	60171834	T	T+	0.167 (-0.18 – 0.52)
*PICALM*	rs3851179	11	86157598	T	C/C	0.098 (-0.23 – 0.42)
*SORL1*	rs11218343	11	121564878	C	T/T	0.214 (-0.68 – 1.10)
*KL*	rs9536314	13	33054001	VS	VS+	0.160 (-0.20 – 0.52)
*FERMT2*	rs17125944	14	52933911	C	C+	0.277 (-0.15 – 0.71)
*SLC24A4*	rs10498633	14	92460608	T	T+	0.110 (-0.21 – 0.43)
*ABCA7*	rs3764650	19	1046521	G	G+	0.062 (-0.34 – 0.47)
*CD33*	rs3865444	19	51224706	A	A+	0.082 (-0.24 – 0.40)
*CASS4*	rs7274581	20	56443204	C	T/T	0.080 (-0.36 – 0.52)
*COMT*	rs4680	22	19963748	Met	Met+	0.098 (-0.26 – 0.45)


*Location and risk information on the 27 genetic variants included in the calculation of the PRS. Minor alleles for SNPs based on published 1000 Genomes Phase 3 European allele frequencies(Genomes Project et al., 2015). Effect sizes [Cohen’s *d* and 95% Confidence Interval (CI)] are presented with their accompanying risk genotype as per calculation in AIBL. SNP, single nucleotide polymorphism; AIBL, Australian Imaging, Biomarkers and Lifestyle Study of Aging.*

### Statistical Analysis

Rstudio (RStudio Team 2015) Version 0.98.1103 for Macintosh was used for all statistical analyses (RStudio Team, 2015). 226 Aβ*^high^* CN older adults were randomly split using the “sample” function of the R “base” package, creating the reference (*n* = 151) and test (*n* = 75) samples sets. Means/counts and standard deviations/percentages for demographic variables for the reference and test groups were then determined. To ensure the reference and test samples were not significantly different in demographic measures analysis of covariance (ANOVA; age, premorbid IQ, depressive symptoms) and chi-squared tests (gender, years of education, *APOE* ε4+ve) were performed.

Calculation of an individual’s *em*PRS was by the summation of each SNP’s effect size for the risk allele, if the risk allele is present. This can be represented as *em*PRS = ∑ RA_*n*_(d_*n*_); where *n* is each individual SNP, *RA* is the presence (1) or absence (0) of risk allele of *n*, and *d* is the calculated effect size for the risk allele of *n*. To calculate *d*, changes in verbal episodic memory performance for individual participants in the reference sample (*n* = 151) were calculated for each SNP using random intercepts linear mixed effects (LME) models, as implemented in the “nlme” package in R, in a dominant model for the minor allele. The resultant mean and standard deviation of the β-coefficient, for the verbal episodic memory slopes, were used to determine the effect sizes for all SNPs (Table 1) using the “effsize” package in R. The resultant effect sizes were then used to assign the risk genotype for each variant. Individual sample *em*PRSs were then calculated by summing the effect sizes if the assigned risk genotypes were present. We have previously reported that the utility of PRSs for prediction of cognitive decline are dependent on the inclusion of *APOE* genotype in their construction (Porter et al., 2018b). As such we wished to determine whether the utility of the *em*PRS defined in this study was likewise dependent on the inclusion of *APOE*. To this end each individual in the test sample had two *em*PRSs calculated. The first included *APOE* (*em*PRS^$\bar{c}$*APOE*^) whilst the second excluded *APOE* (*em*PRS^$\bar{s}$*APOE*^).

Random intercepts LME models were used to assess associations between both *em*PRS^$\bar{c}$*APOE*^ and *em*PRS^$\bar{s}$*APOE*^ and cognitive performance in the test sample (*n* = 75). Modeling was again performed using the “nlme” R package. For all analyses, the cognitive composite scores were included as dependent variables, *em*PRS × Time interactions as fixed factors, and participant baseline scores as random factors. Additionally, those analyses investigating associations with AIBL-PACC performance included age as a covariate. Further, the test sample was then stratified based on the number of *APOE* ε4 alleles carried, and the model described above was used to determine associations of the *em*PRS^$\bar{s}$*APOE*^ in each group. All models were graphically represented as baseline or change in composite score given *em*PRS, with error shading signifying *em*PRS dependent standard error.

## Results

### SNP Information, PRS Calculation and Demographics

A total of 27 SNPs were identified and selected for inclusion into the current *em*PRSs. Effect sizes were calculated for the selected SNPs, based on their discriminatory effect on decline in verbal episodic memory in the reference cohort. These are presented with corresponding 95% confidence intervals in Table 1. The effect sizes ranged from *APOE*, with the largest effect size (*d* = 0.472), to *BIN1* with the smallest (*d* = 0.003). These effect sizes were then used for weighting of each SNP in the calculated *em*PRS in the test cohort. No significant differences were identified between the reference (*n* = 151) and test (*n* = 75) samples for the baseline demographic measures (Table 2).

**Table 2:** Demographic information.

		Overall (*n* = 226)	Reference (*n* = 151)	Test (*n* = 75)	*P*
Age, mean (SD), years		72.24 (6.57)	72.02 (6.48)	72.69 (6.78)	0.482
Female, No. (%)		116 (51.33)	77 (50.99)	39 (52.00)	0.999
Years of Education, No. (%)	0–8 years	20 (8.93)	10 (6.67)	10 (13.51)	0.221
	9–12 years	84 (37.50)	55 (36.67)	29 (39.19)	
	13–15 years	50 (22.32)	33 (22.00)	17 (22.97)	
	15+ years	70 (31.25)	52 (34.67)	18 (24.32)	
Premorbid IQ (FSIQ), mean (SD)		108.06 (7.46)	108.01 (7.68)	108.18 (7.05)	0.872
Depressive Symptoms (GDS), mean (SD)		1.01 (1.23)	1.08 (1.29)	0.87 (1.11)	0.318
*APOE* ε4 carriage, No. (%)		95 (42.04)	61 (40.40)	34 (45.33)	0.572


*Baseline demographic and clinical characteristics for all cognitively normal older adults with high Aβ (Aβ*^*high*^*) in the AIBL study, and stratified by the reference (*n* = 152) and test (*n* = 75) cohorts utilized in the current study. Values represented as mean/count (standard deviation/percentage). Significance of statistical difference between reference and test cohorts represented by *p*-values. GDS, Geriatric Depression Scale; FSIQ, Wechsler Adult Intelligence Scale-Third Edition (WAIS-III) Full Scale Intelligence Quotient.*

### *em*PRS^$\bar{c}$*APOE*^ and *em*PRS^$\bar{s}$*APOE*^ Are Associated With Longitudinal Cognition in Aβ^high^ CN Older Adults

**FIGURE 1:**
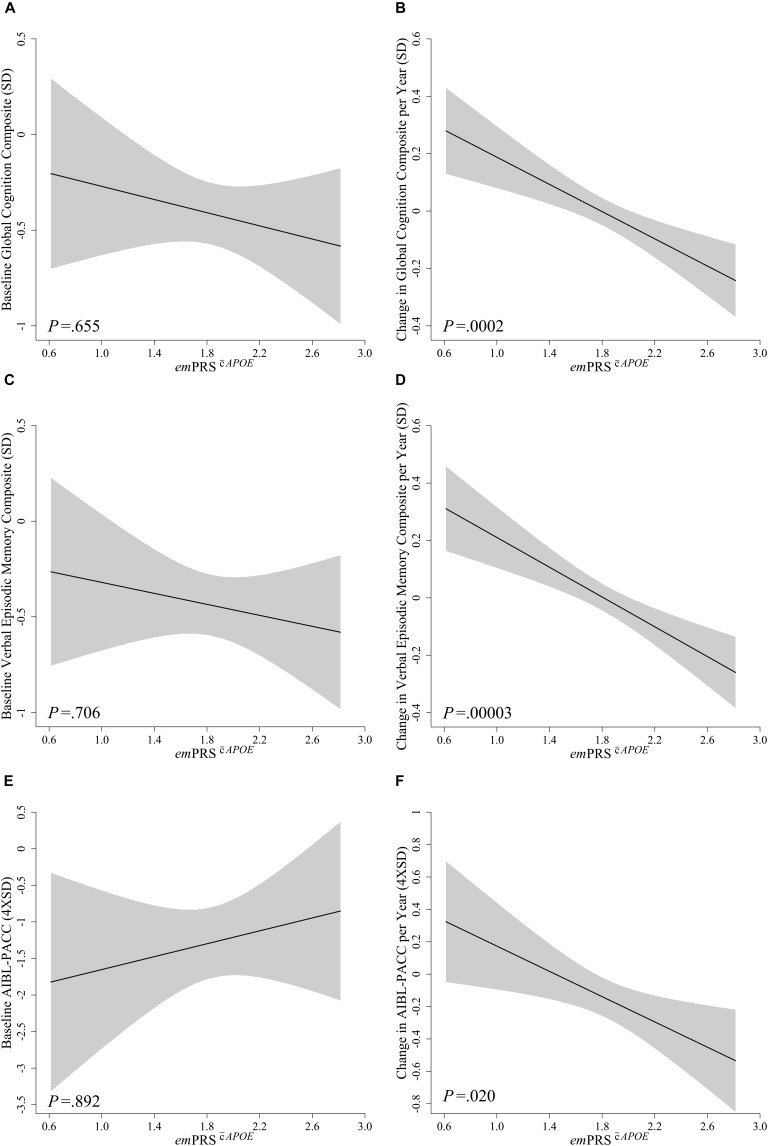
Association between *em*PRS^$\bar{c}$*APOE*^ and baseline and longitudinal change in cognition in Aβhigh CN older adults. Association between *em*PRS^$\bar{c}$*APOE*^ and baseline **(A,C,E)** and longitudinal change **(B,D,F)** in composite measures of cognition including a statistically driven global composite **(A,B)**, verbal episodic memory composite **(C,D)**, and Pre-Alzheimer’s Cognitive Composite (AIBL-PACC; **E,F**) in cognitively normal (CN) older adults with high Aβ (Aβ^high^; *n* = 75). AIBL-PACC controlled for age. Shaded regions represent *em*PRS dependent standard error. *em*PRS^*APOE*^, cognition polygenic risk score with APOE genotype.

**FIGURE 2:**
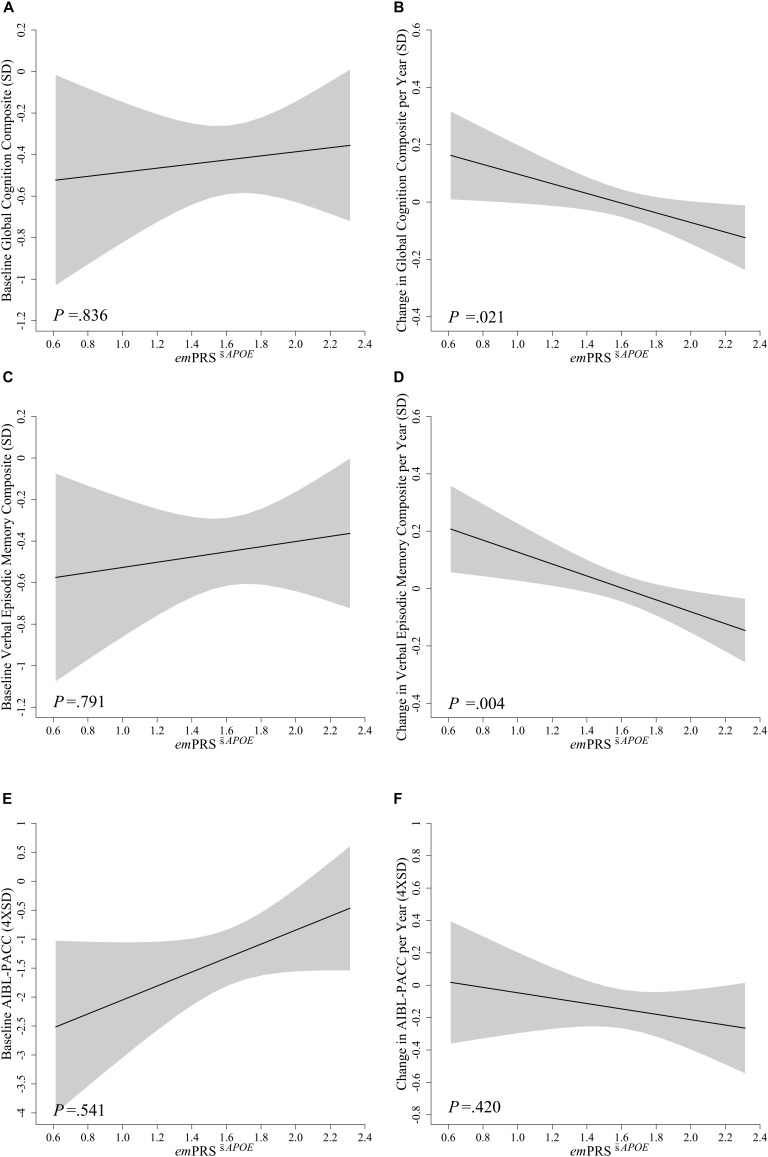
Association between *em*PRS^$\bar{s}$*APOE*^ and baseline and longitudinal change in cognition in Aβ^high^ CN older adults. Association between *em*PRS^$\bar{s}$*APOE*^ and baseline **(A,C,E)** and longitudinal change **(B,D,F)** in composite measures of cognition including a statistically driven global composite (A,B), verbal episodic memory composite **(C,D)**, and Pre-Alzheimer’s Cognitive Composite (AIBL-PACC; **E,F**) in cognitively normal (CN) older adults with high Aβ (Aβ^high^; *n* = 75). AIBL-PACC controlled for age. Shaded regions represent emPRS dependent standard error. *em*PRS^$\bar{s}$*APOE*^, cognition weighted polygenic risk score without APOE genotype.

No significant associations were observed between *em*PRS^$\bar{c}$*APOE*^ or *em*PRS^$\bar{s}$*APOE*^ and performance at baseline for global cognition (*em*PRS^$\bar{c}$*APOE*^: -0.172, *p* = 0.655; *em*PRS^$\bar{s}$*APOE*^: 0.098, *p* = 0.836), verbal episodic memory (*em*PRS^$\bar{c}$*APOE*^: -0.144, *p* = 0.706; *em*PRS^$\bar{s}$*APOE*^: 0.125, *p* = 0.791) or the AIBL-PACC (*em*PRS^$\bar{c}$*APOE*^: 0.143, *p* = 0.892; *em*PRS^$\bar{s}$*APOE*^: 0.786, *p* = 0.541) (Figures 1, 2). The *em*PRS^$\bar{c}$*APOE*^ was significantly associated with longitudinal cognitive performance as measured by global cognition (-0.237, *p* = 0.0002), verbal episodic memory (-0.259, *p* = 0.00003) and the AIBL-PACC (-0.381, *p* = 0.020) (Figure 1). Specifically, in the test cohort, as the *em*PRS^$\bar{s}$*APOE*^ increased the rate of decline in cognitive outcomes worsened.

To assess whether these associations were dependent upon the inclusion of *APOE* in the calculation, the *em*PRS was calculated excluding *APOE.* This derived *em*PRS, *em*PRS^$\bar{s}$*APOE*^, was likewise observed to be significantly associated with longitudinal global cognition (-0.169, *p* = 0.021) and verbal episodic memory (-0.208, *p* = 0.004) performance, albeit to a reduced extent (Figure 2). However, it was no longer associated with decline on the AIBL-PACC (-0.152, *p* = 0.420). As was the case when investigating *em*PRS^$\bar{c}$*APOE*^, an increase in *em*PRS^$\bar{s}$*APOE*^ was significantly associated with a concomitant increased rate of cognitive decline.

### *em*PRS^$\bar{s}$*APOE*^ Is Associated With Longitudinal Cognition in Aβ^high^ CN Older Adults, When Stratified by *APOE ε4* Carriage

After stratification of the test sample into groups based on the carriage of the *APOE* ε4 allele, association between the *em*PRS^$\bar{s}$*APOE*^ and cognition was evaluated separately. Significant associations between the *em*PRS^$\bar{s}$*APOE*^ and longitudinal measures of verbal episodic memory and global cognition were observed irrespective of *APOE* ε4 status (Table 3). However, the *em*PRS^$\bar{s}$*APOE*^ association with decline in the AIBL-PACC was limited to *APOE* ε4 carriers. Finally, no associations were observed in any groups when investigating baseline levels of cognition.

**Table 3:** Association between *em*PRS^$\bar{s}$*APOE*^ and baseline and longitudinal change in cognition in Aβ^high^ CN older adults.

	α	*P*	β	*P*
***APOE*ε4 non-carrier (*n* = 41)**				
Global	0.210	0.619	-0.145	0.044
Verbal Episodic Memory	0.242	0.575	-0.185	0.011
AIBL-PACC	0.531	0.708	0.086	0.645
***APOE*ε4 carrier (*n* = 34)**				
Global	0.032	0.972	-0.329	0.028
Verbal Episodic Memory	0.047	0.958	-0.363	0.013
AIBL-PACC	1.477	0.522	-0.821	0.034


*Mean slopes for models assessing the association between *em*PRS and baseline (α) and longitudinal (β) change in cognitive composite measures in all cognitively normal (CN) older adults with high Aβ (Aβ*^*high*^; n* = 75), and by carriage of the *APOE*ε4 allele. AIBL-PACC controlled for age. *em*PRS^$\bar{s}$*APOE*^, cognition weighted polygenic risk score without *APOE* genotype.*

## Discussion

This study describes a *em*PRS developed by weighting AD risk and cognition associated genetic variants by their effect on decline in verbal episodic memory in a cohort defined in terms of pre-clinical AD by reference to neocortical Aβ PET imaging. The *em*PRSs were calculated in the test sample by the summation of gene variant effect sizes calculated with respect to decline in episodic memory in the reference sample. In the test sample no associations were observed between the *em*PRSs and baseline levels of cognition, however, associations with longitudinal performance were statistically significant. The *em*PRS^$\bar{c}$*APOE*^ was significantly associated with decline in global cognition, verbal episodic memory and the AIBL-PACC (Table 3 and Figure 1). While these associations were strongest when *APOE* was included they were not dependent on the inclusion of *APOE* ε4, as associations with verbal episodic memory and global cognition were still observed in the *em*PRS^$\bar{s}$*APOE*^ (Table 3 and Figure 2). Further evidence that the score developed was not purely dependent on *APOE* was provided when the participants were stratified by carriage of the *APOE*ε4 allele. This analysis showed that the *em*PRS^$\bar{s}$*APOE*^ was significantly associated with verbal episodic memory and global cognition in both carriers and non-carriers of the *APOE*ε4 allele.

Previous studies have observed significant associations between PRSs weighted by a measure of AD risk and cognitive performance in a number of different domains (Sabuncu et al., 2012; Carrasquillo et al., 2015; Andrews et al., 2016; Louwersheimer et al., 2016; Marden et al., 2016; Mormino et al., 2016). Further, these associations have been reported in both cognitively normal individuals (Sabuncu et al., 2012; Andrews et al., 2016; Marden et al., 2016) and those who had already developed AD (Carrasquillo et al., 2015). Few studies have investigated the utility of PRSs independent of *APOE* genotype or have reported no associations when *APOE* was excluded, similar to our previous study (Porter et al., 2018b). However, two studies have observed significant associations between clinical and cognitive outcomes and PRS independent of *APOE* (Mormino et al., 2016; Desikan et al., 2017). The major difference in these studies was that they either involved a phenotype correction within the PRS calculation (Desikan et al., 2017) or significantly extended the number of SNPs included in the PRS (Mormino et al., 2016). Unlike the current study, neither of these prior studies used a phenotype weighting system for the development of an *APOE* independent PRS with a reduced number of genetic variants. To the best of our knowledge, this is the first PRS developed through weighting by a cognitive phenotype and specifically with the aim of predicting decline in a preclinical AD cohort.

The effect size for *APOE* observed in the study is similar to that reported previously in this cohort. One contrast is that in the previous study it was over a shorter duration (4.5 years) and used a learning/working memory composite derived from the online Cogstate Brief Battery (Lim et al., 2015b). In this study there was no obvious disparity in the calculated effect sizes between the *a priori* cognition associated variants or GWAS-derived AD risk variants. This lack of disparity supports both our notion of phenotype-specific effect size driven PRSs, as well as the importance of combining both cognition and AD risk associated variants. In addition, after excluding *APOE*, the variants with the 3 largest effect sizes in this study were rs190982 [Myocyte-specific enhancer factor 2C (*MEF2C*)], rs9271192 (*HLA* cluster), and rs17125944 [Fermitin family homolog 2 (*FERMT2*)]. While these variants were included due to their previous associations with AD risk, they have been associated (albeit inconsistently) with cognitive performance and/or decline. In one study, *MEF2C* was associated with general cognitive function whilst neither *FERMT2* or genes in the *HLA* cluster were associated (Davies et al., 2015). In a further study, analysis of the same variants in the current study revealed a trend toward association of *MEF2C* (rs190982) whilst the *HLA* cluster (rs9271192), and *FERMT2* (rs17125944) were not significant (Nettiksimmons et al., 2016). However, in the same study aggregate associations of SNPs within the *MEF2C* and *HLA* loci were associated with cognitive decline (Nettiksimmons et al., 2016). The protein products of these genes do have functions in neuronal homeostasis and plasticity that would indicate they could be associated with maintaining cognitive functioning. *MEF2C* is reported to be involved in neurogenesis (Li et al., 2008), whilst its deletion in the CNS of mice impairs hippocampal-dependent learning and memory (Barbosa et al., 2008) and peripheral mRNA expression has been reported to correlate with memory performance in a Japanese sample (Sao et al., 2017). The *HLA* cluster has important roles in the immune response, and *FERMT2* maintains cellular structures including neuronal cells.

It is acknowledged that the current study has several limitations. First, AIBL study participants generally have higher levels of education, which may not be representative of the broader community (Ellis et al., 2009). Second, the current study represents a majority Caucasian population and results may differ based on ethnicity. Finally, small sample sizes in the resulting reference and test cohorts may have influenced the results reported. This is evidenced by differences in the risk alleles of certain SNPs observed in this study, compared with those previously reported. In addition to these limitations, several strengths of the AIBL study may impact replication studies. Firstly, the calculation of effect sizes for PRS weighting a combined group measure of verbal episodic memory performance over 7.5 years was utilized. It is possible that studies of shorter duration could yield differing results. Second, the use of a composite measure of verbal episodic memory is a further strength of the study, the same or comparable neuropsychological tests are likely to be required to calculate appropriate cognitive composite scores for validation purposes. For this reason, the replication of the methods and results reported here in large, comparably comprehensive studies are warranted to validate the clinical utility of this *em*PRS.

## Conclusion

The study detailed above describes a *em*PRS for the prediction of rates of cognitive decline in cognitively normal older adults at risk for AD. It shows that the *em*PRS is able to predict rates of cognitive decline in domains typically affected in the preclinical stages of AD. Further, this is the first PRS calculated with a conservative number of genetic variants to be associated with longitudinal cognition in the absence of *APOE.* The reported *em*PRS has utility in those individuals carrying no or one copy of the *APOE* ε4 allele. The results presented here provide evidence which support the further evaluation and use of endophenotype weightings in PRS development rather than the standard AD risk weightings that demonstrate inconsistent utility. The methodology and results should be validated in similarly well-characterized cohorts with comparable phenotypic and longitudinal data.

## Data Availability Statement

All data and samples used in this study are derived from the Australian Imaging, Biomarkers and Lifestyle (AIBL) Study of Ageing. AIBL data can be accessed through an Expression of Interest procedure, for more information please see https://aibl.csiro.au/awd.

## Author Contributions

TP contributed to acquisition of genetic data, statistical analysis, interpretation of findings, and drafting the manuscript. SB contributed to specific study concept and design, study supervision, statistical analysis, interpretation of findings, and revising the manuscript. GS and PM contributed to AIBL study design, obtaining funding, interpretation of findings, and revising the manuscript. YL contributed to interpretation of findings and revising the manuscript. LM and MP contributed to acquisition of genetic data. DA, CM, CR, and RM contributed to AIBL study design, obtaining funding, and revising the manuscript. SR-S and KT contributed to revising the manuscript. DG and GV contributed to study supervision and revising the manuscript. VV contributed to current study concept and design, obtaining funding, study supervision, acquisition of data, interpretation of findings, and revising the manuscript. SL contributed to current study concept and design, obtaining funding, study supervision, acquisition of data, interpretation of findings, and drafting of the manuscript. All authors read and approved the final manuscript.

## Conflict of Interest Statement

CM is an advisor to Prana Biotechnology Ltd., and a consultant to Eli Lilly. PM is a full-time employee of Cogstate Ltd. YL reports serving as a scientific consultant to CogState Ltd., Biogen, and Lundbeck. CR has served on scientific advisory boards for Bayer Pharma, Elan Corporation, GE Healthcare, and AstraZeneca, has received speaker honoraria from Bayer Pharma and GE Healthcare, and has received research support from Bayer Pharma, GE Healthcare, Piramal Lifesciences and Avid Radiopharmaceuticals. VV served as a consultant for Bayer Pharma, and received research support from a NEDO grant from Japan. The remaining authors declare that the research was conducted in the absence of any commercial or financial relationships that could be construed as a potential conflict of interest.
